# Is *Wolbachia* a sustainable component for dengue control?

**DOI:** 10.1590/0074-02760250268

**Published:** 2026-06-01

**Authors:** Narayanasamy Sivagnaname, Sivagnaname Yuvarajan, Raja Mahendran

**Affiliations:** 1 Indian Council of Medical Research, Vector Control Research Centre, Puducherry, India; 2 Sri Mankula Vinayagar Medical College and Hospital, Puducherry, India; 3 International Pest Business Consultant, Sydney, Australia

**Keywords:** Wolbachia, dengue control, cytoplasmic incompatibility, mechanical transmission, surveillance

## Abstract

Wolbachia-based vector control has emerged as one of the most innovative biological interventions in contemporary dengue prevention, offering an ecologically grounded alternative to insecticide-dependent strategies. By exploiting cytoplasmic incompatibility (CI) and strain-specific viral interference, Wolbachia pipientis-transinfected Aedes aegypti (Linnaeus, 1762) mosquitoes can reduce viral transmission, as demonstrated by the Wolbachia-based field trials demonstrating proof of principle. However, the long-term sustainability of this approach remains uncertain. Accumulating field evidence indicates that Wolbachia persistence and epidemiological impact are highly context-dependent, shaped by mosquito fitness, strain-host compatibility, climatic sensitivity, urban ecology and operational continuity. Experiences from Vietnam, Thailand and parts of Brazil illustrate that stable introgression cannot be assumed and may decline when releases are interrupted or ecological conditions are unfavourable. Operational constraints — including mass-rearing quality, dispersal limitations, surveillance intensity and financial costs — pose additional scalability challenges. This perspective critically evaluates whether Wolbachia can function as a sustainable dengue control strategy across diverse endemic settings. We argue that Wolbachia should not be framed as a self-sustaining or stand-alone solution, but rather as a maintenance-dependent, context-sensitive intervention whose effectiveness relies on ecological tailoring, thermotolerant strains, robust post-release surveillance and integration within adaptive integrated vector management (IVM) frameworks. When deployed judiciously alongside complementary control measures, Wolbachia has the potential to become an important — though not exclusive — pillar of long-term dengue control.

Dengue continues to represent a major global public health challenge, placing nearly half of the world’s population at risk.^1^ Its sustained expansion reflects the interaction of climate variability, rapid urbanisation, population mobility and the exceptional ecological adaptability of *Aedes aegypti* (Linnaeus, 1762).^2^ ^,^ ^3^ In the absence of broadly effective antivirals, a universally protective vaccine or reliable early warning systems, vector control remains the principal strategy for dengue prevention.^3^ However, conventional insecticide-based approaches are increasingly constrained by cryptic breeding habitats, outdoor resting behaviour and the rapid emergence of insecticide resistance,^4^ underscoring diminishing returns of traditional control paradigms.

In this context, *Wolbachia pipientis* — an intracellular bacterium naturally absent in *Ae. aegypti* — has been proposed as an alternative biological intervention. Transinfection with *W. pipientis* can reduce arboviral replication and induce cytoplasmic incompatibility (CI), enabling population replacement with mosquitoes of reduced vector competence or, in some settings, population suppression.^5^ ^,^ ^6^ ^,^ ^7^ ^,^ ^8^ Early field deployments demonstrated technical feasibility, yet outcomes have been heterogeneous across ecological and urban contexts, challenging assumptions of universal effectiveness and long-term self-sustainability.^9^

Evidence increasingly indicates that successful establishment depends on sustained infection densities, strain-host compatibility, environmental stability and intensive release strategies, compounded by the limited dispersal range of *Ae. aegypti*.^6^ Operational scalability is further constrained by fitness costs, mass-rearing and sex-separation requirements, regulatory oversight and long-term financial commitments. Social acceptance and community engagement remain decisive determinants of programme continuity.^7^ Variability in performance across settings highlights the vulnerability of *Wolbachia*-based strategies to ecological, climatic and programmatic disruption.

Importantly, even under optimal conditions, *Wolbachia* interventions cannot address all transmission pathways nor mitigate the inherently cyclical and multifactorial nature of dengue epidemiology. Their public health value is therefore context-dependent and contingent on integration within adaptive, evidence-driven integrated vector management (IVM) frameworks rather than deployment as a stand-alone solution.^8^

This perspective critically examines the biological assumptions, operational constraints and epidemiological uncertainties surrounding *Wolbachia*-based dengue control, questioning whether it can realistically function as a sustainable intervention or whether its promise remains bounded by structural and ecological limitations.

## Ecological and operational realities

The deployment of *Wolbachia*-infected *Ae. aegypti* has generated optimism for dengue control, yet long-term success depends on ecological nuance and operational precision. Although *Ae. aegypti* was historically considered naturally *Wolbachia*-free, recent evidence reveals low-density resident strains, including wAlbB-like variants, in wild populations.^10^ ^,^ ^11^ These cryptic infections introduce complexity: interactions between resident and introduced strains may alter CI, maternal transmission fidelity and viral blocking efficiency.^12^ Rigorous pre-release molecular screening and phylogenetic characterisation are therefore essential.

Transmission dynamics add further complexity. Dengue exhibits cyclical peaks every two-three years, driven by fluctuating herd immunity, serotype shifts and climate variability, particularly El Niño-Southern Oscillation patterns.^13^ ^,^ ^14^ Post-release reductions may coincide with natural troughs, potentially inflating perceived impact. Only sustained multi-year entomological and epidemiological monitoring can disentangle intervention effects from background variability.

Even in *Wolbachia*-dominated populations, dengue may persist via overlooked pathways — including rare vertical transmission or mechanical transfer.^15^ ^,^ ^16^ Though likely minor, these routes reinforce that *Wolbachia* cannot operate in isolation. Complementary strategies — environmental management, community participation and enhanced surveillance — remain indispensable.

Operational performance depends critically on mosquito fitness. Laboratory rearing may impose trade-offs affecting longevity, fecundity and mating competitiveness.^17^ ^,^ ^18^ ^,^ ^19^ For replacement strategies to succeed, infected females must sustain high maternal transmission fidelity, while males must induce robust CI without substantial fitness penalties.^20^ ^,^ ^21^ ^,^ ^22^ Field assessments of dispersal, survival and reproductive performance are thus essential to prevent ecological bottlenecks that could undermine establishment.

## Balancing suppression, replacement, and integration

*Wolbachia*-based interventions operate along two principal trajectories: mosquito population suppression and population replacement.

Suppression strategies rely on the release of *W. pipientis*-infected males that induce CI when mating with uninfected females, resulting in embryonic lethality and rapid reductions in mosquito density.^5^ ^,^ ^23^ Because only males are released, there is no increased risk of disease transmission. However, suppression effects are inherently transient; once releases cease, mosquito populations may rebound, particularly in high-transmission urban settings.

In contrast, population replacement strategies release both males and females, enabling *Wolbachia* to establish maternally inherited infections in wild *Ae. aegypti* populations.^18^ ^,^ ^21^ ^,^ ^24^ Replacement does not primarily reduce mosquito density but instead lowers vector competence by limiting dengue virus (DENV) replication and dissemination. Once *Wolbachia* reaches high prevalence, the intervention may persist without continuous releases. This apparent durability positions replacement as transformative, although establishment depends heavily on ecological suitability, strain-host compatibility and environmental stability.

An important dimension of replacement is its influence on vertical transmission. By reducing viral replication within mosquito ovaries, *Wolbachia* may disrupt transovarial transmission that otherwise allows DENV persistence during inter-epidemic periods.^18^ ^,^ ^24^ ^,^ ^25^ Nevertheless, interference varies according to strain, mosquito genetic background and climatic conditions, underscoring the need for field-validated assessments before large-scale deployment.

Mechanistically, *Wolbachia*-mediated viral blocking involves immune priming, competition for host cellular resources and disruption of viral replication complexes.^26^ ^,^ ^27^ ^,^ ^28^ Although these mechanisms effectively limit positive-sense RNA viruses such as DENV, ecological and evolutionary consequences require continued monitoring.

Integrating suppression and replacement-tailored to local epidemiology-offers a layered strategy: suppression for rapid outbreak control and replacement for longer-term reduction in vector competence. When embedded within adaptive IVM, these approaches enhance programme resilience and sustainability.

## The conditional success of *Wolbachia*: genetic, ecological, and epidemiological dimensions

CI forms the biological engine of *Wolbachia*-based control.^22^ ^,^ ^25^ Yet CI effectiveness is not universal; it depends on compatibility between released and local mosquito genotypes.^6^ ^,^ ^29^ ^,^ ^30^ ^,^ ^31^ Genetic mismatches can slow spread, weaken viral blocking or compromise persistence. Matching release strains with local populations is therefore essential for successful introgression.

Environmental factors further modulate outcomes. Temperature, humidity and rainfall profoundly influence *Wolbachia* density, mosquito fitness and DENV transmission dynamics.^12^ ^,^ ^32^ ^,^ ^33^ ^,^ ^34^ Elevated temperatures may reduce *Wolbachia* density, diminish viral-blocking efficiency and lower maternal transmission fidelity.^17^ ^,^ ^35^ Simultaneously, warmer conditions accelerate the extrinsic incubation period of DENV, potentially offsetting gains achieved through *Wolbachia* deployment. These interactions illustrate that impact is geographically variable and environmentally contingent.

Integrative eco-epidemiological monitoring is therefore indispensable. Surveillance must encompass mosquito density, *Wolbachia* prevalence, viral circulation, climatic trends and human case incidence.^28^ Without sustained multi-year data, programmes risk attributing natural epidemiological troughs to intervention success.

These realities frame *Wolbachia* not as a universal solution but as a context-sensitive innovation. Success depends on harmonising genetic compatibility, strain robustness, field fitness and climatic resilience. Adaptive governance and evidence-driven refinement remain central to durability.

## Global perspectives on *Wolbachia*-based dengue control

Field deployments provide the strongest empirical evidence of *Wolbachia*’s potential. In northern Queensland, Australia, phased releases of the wMel strain achieved near-fixation (> 90%) and sustained prevalence for over a decade without further releases.^21^ This success was supported by rigorous surveillance, community engagement and operational continuity.

In Indonesia, the cluster-randomised AWED trial in Yogyakarta demonstrated a 77% reduction in laboratory-confirmed dengue and an 86% decline in hospitalisations over two years following *Wolbachia* establishment.^6^ These findings provided robust epidemiological validation under controlled trial conditions.

Brazilian deployments in Rio de Janeiro and Niterói demonstrated feasibility in large, complex urban settings.^5^ ^,^ ^28^ Moderate-to-high establishment was achieved despite infrastructural and socio-economic challenges, with reductions in dengue incidence approaching 69% in some areas.

However, experiences from Vietnam and Thailand illustrate operational and ecological constraints. In Nha Trang, *Wolbachia* prevalence fluctuated seasonally, and epidemiological impact was limited.^36^ ^,^ ^37^ In Thailand, hybrid suppression-replacement strategies struggled to maintain stable infection frequencies.^38^ Thermal stress, reduced fitness of laboratory-reared mosquitoes and complex urban ecologies limited persistence.^12^ ^,^ ^17^ ^,^ ^33^

These comparative insights are synthesised in Table, which summarises success factors, limiting determinants and cross-cutting residual risks across diverse settings.

**TABLE:** Key global insights into *Wolbachia*-based dengue control: successes, challenges and residual risks

Dimension	Success factors (Australia, Indonesia, Brazil)	Limiting factors (Vietnam, Thailand, others)	Residual risks (Cross-cutting)
Epidemiological impact	10 years without outbreaks (Australia); 77% reduction in confirmed cases, 86% decline in hospitalisations (Indonesia); up to 69% incidence reduction (Brazil).	Inconsistent prevalence; weak or no measurable epidemiological signal despite releases.	Possible persistence of dengue through silent or residual transmission pathways.
Operational strengths	Phased releases, rigorous monitoring, strong community engagement; large-scale releases feasible (Brazil: >5 million mosquitoes).	Limited release coverage, hybrid strategies with unstable *Wolbachia* frequencies, logistical challenges.	Dependence on continuous surveillance; operational fatigue in long-term programs.
Strain performance	Stable wMel establishment in moderate climates, high maternal transmission fidelity.	Reduced *Wolbachia* density at high temperatures and diminished viral-blocking under heat stress.	Strain-specific vulnerabilities; need for thermally robust and high-fitness variants.
Mosquito fitness	Sustained maternal transmission, effective cytoplasmic incompatibility (CI) induction, stable prevalence.	Fitness costs in mass-reared mosquitoes: reduced survival, fecundity, delayed development, poor male competitiveness.	Risk of ecological bottlenecks undermining long-term establishment.
Community & social acceptance	High trust and participation (Australia, Indonesia); gradual acceptance in Brazil through targeted outreach.	Initial scepticism, slower adoption in areas with fragmented social trust.	Sustained engagement is essential; misinformation or distrust could reverse gains.
Ecological & climatic context	Moderate climates with stable *Wolbachia* persistence.	Seasonal rainfall, urban ecological complexity, and high ambient temperatures disrupt prevalence.	Climate change may destabilise *Wolbachia* density and viral-blocking capacity.
Integration with broader control	Complementary to existing vector control programs; strengthens integrated vector management.	Over-reliance on *Wolbachia* without supportive measures weakens outcomes.	Residual reliance on insecticides, surveillance, and community actions is inevitable.

## Silent sparks: mechanical transmission and residual risk

While *Wolbachia* blocks biological transmission of DENV, it does not address mechanical transmission (MT). MT occurs when infectious viral particles are transferred between hosts via contaminated mouthparts during interrupted feeding or multiple probing events.^39^ ^,^ ^40^ ^,^ ^41^ ^,^ ^42^ Unlike biological transmission, MT is immediate and bypasses intracellular viral replication, rendering *Wolbachia*-mediated blocking irrelevant.

The epidemiological significance of MT remains debated. Experimental evidence confirms plausibility.^39^ ^,^ ^41^ ^,^ ^43^ but its contribution to sustained epidemics is likely limited compared with classical biological transmission. Nonetheless, in densely populated urban environments with high biting rates, MT could act as a sporadic ignition mechanism, potentially explaining isolated cases within *Wolbachia*-dominated populations.

Addressing residual risk requires integrated strategies. *Wolbachia*-based replacement must be complemented by measures that reduce biting pressure and overall mosquito density. Environmental management, adult suppression tools and behavioural surveillance remain critical. Recognising MT reinforces the principle that *Wolbachia* functions most effectively within a broader, adaptive IVM framework.

## Discussion and conclusion

*Wolbachia*-based interventions represent a significant paradigm shift in dengue control, transitioning from chemical suppression toward biologically mediated modification of vector competence. Field evidence from Australia, Indonesia and Brazil demonstrates that, under favourable ecological and operational conditions, near-fixation of *Wolbachia* can be achieved and sustained, resulting in substantial reductions in dengue transmission.^5^ ^,^ ^6^ ^,^ ^21^

Conversely, heterogeneous outcomes in Vietnam, Thailand and complex urban contexts highlight ecological and operational fragility.^36^ ^,^ ^37^ ^,^ ^38^ Thermosensitivity of key strains, reduced fitness of mass-reared mosquitoes and climatic variability may undermine persistence. Emerging data further suggest that long-term introgression may decline when operational support weakens.^9^

Residual transmission pathways — including vertical and mechanical transmission^39^ ^,^ ^40^ ^,^ ^41^ ^,^ ^42^ ^,^ ^43^ and dengue’s inherently cyclical epidemiology necessitate prolonged, multi-year evaluation before claims of durable elimination can be substantiated.

Collectively, current evidence indicates that *Wolbachia* is neither a universal remedy nor an inherently self-sustaining intervention. Rather, it is a powerful but conditional tool whose long-term success depends on genetic compatibility, ecological resilience, operational continuity and integration with complementary vector control strategies. When embedded within adaptive, evidence-driven IVM frameworks, *Wolbachia* can substantially reduce dengue transmission risk. Its enduring value lies not in replacing existing tools but in strengthening them — advancing dengue control toward a systems-based, sustainable prevention paradigm.

## Data Availability

No new data were generated or analysed in this study. All information is derived from previously published literature.
